# Novel Therapeutic Potentials of Taxifolin for Amyloid-β-associated Neurodegenerative Diseases and Other Diseases: Recent Advances and Future Perspectives

**DOI:** 10.3390/ijms20092139

**Published:** 2019-04-30

**Authors:** Masashi Tanaka, Satoshi Saito, Takayuki Inoue, Noriko Satoh-Asahara, Masafumi Ihara

**Affiliations:** 1 Department of Physical Therapy, Health Science University, 7187 Kodachi, Fujikawaguchiko-machi, Minamitsuru-gun, Yamanashi 401-0380, Japan; 2 Department of Endocrinology, Metabolism, and Hypertension Research, Clinical Research Institute, National Hospital Organization Kyoto Medical Center, 1-1 Fukakusa Mukaihata-cho, Fushimi-ku, Kyoto 612-8555, Japan; taka2015.www@gmail.com (T.I.); nsatoh@kuhp.kyoto-u.ac.jp (N.S.-A.); 3 Department of Neurology, National Cerebral and Cardiovascular Center, 5-7-1 Fujishiro-dai, Suita, Osaka 565-8565, Japan; ihara@ncvc.go.jp; 4 Research Fellow of Japan Society for the Promotion of Science, 5-3-1 Kojimachi, Chiyoda-ku, Tokyo 102-0083, Japan

**Keywords:** cerebral amyloid angiopathy, Alzheimer’s disease, amyloid-β fibril formation, taxifolin

## Abstract

Amyloid-β (Aβ) has been closely implicated in the pathogenesis of cerebral amyloid angiopathy (CAA) and Alzheimer’s disease (AD), the major causes of dementia. Thus, Aβ could be a target for the treatment of these diseases, for which, currently, there are no established effective treatments. Taxifolin is a bioactive catechol-type flavonoid present in various plants, such as herbs, and it exhibits pleiotropic effects including anti-oxidant and anti-glycation activities. Recently, we have demonstrated that taxifolin inhibits Aβ fibril formation in vitro and have further shown that it improves cerebral blood flow, facilitating Aβ clearance in the brain and suppressing cognitive decline in a mouse model of CAA. These findings suggest the novel therapeutic potentials of taxifolin for CAA. Furthermore, recent extensive studies have reported several novel aspects of taxifolin supporting its potential as a therapeutic drug for AD and metabolic diseases with a high risk for dementia as well as for CAA. In this review, we have summarized the recent advances in taxifolin research based on in vitro, in vivo, and in silico approaches. Furthermore, we have discussed future research directions on the potential of taxifolin for use in novel therapeutic strategies for CAA, AD, and metabolic diseases with an increased risk for dementia.

## 1. Introduction

Cerebral amyloid angiopathy (CAA), pathologically characterized by the deposition of amyloid-β (Aβ) within small cerebral arteries, is a major cause of cerebrovascular diseases. Approximately 40% of intracerebral hemorrhage (ICH) cases are associated with moderate or severe CAA in the UK [1]. Currently, there are no established treatments for CAA [2]. Despite great advances in ischemic stroke care, long-term prognosis of ICH remains a cause for concern [3].

We have recently reported that taxifolin, a catechol-type flavonoid with strong anti-oxidant and anti-glycation activities, inhibits Aβ aggregation, thus reducing cerebrovascular Aβ accumulation. This review mainly focuses on the potential of taxifolin as a novel treatment for CAA and other diseases.

## 2. Cerebral Amyloid Angiopathy

### 2.1. Overlapped Pathophysiologies Between Cerebral Amyloid Angiopathy and Alzheimer’s Disease

Aβ accumulates in the cerebral vessel walls, causing smooth muscle cell degeneration, vessel wall thickening, luminal narrowing, and concentric wall splitting (double barreling) [4]. These vascular pathologies are hallmarks of CAA and cause variable degrees of cerebral hemorrhage. In addition to symptomatic intracranial hemorrhage, asymptomatic cerebral microbleeds (CMBs) and superficial siderosis are frequently found on T2* gradient-recalled echo or susceptibility-weighted magnetic resonance imaging [5,6]. Lobar, but not deep, CMBs are especially related to cerebrovascular Aβ accumulation. Thus, multiple, strictly lobar CMBs could be a useful marker for the diagnosis of CAA [7]. CAA also induces ischemic strokes. In addition to macroinfarcts [8], cerebral microinfarctions (CMIs) are associated with cognitive impairment [9]. Reportedly, patients with CAA have a greater number of CMIs than controls [10], and the number occasionally exceeds 1000 [11]. 

CAA plays a pivotal role in the pathogenesis of dementia. CAA is extremely common in sporadic Alzheimer’s disease (AD) [12], suggesting a strong, bidirectional relationship between AD and CAA. Furthermore, CAA increases the odds of AD and is associated with increased cognitive decline [13]. Cognitive changes are likely to be associated with CAA-related pathologies such as cerebral hemorrhage and infarcts. Therefore, preventing cerebrovascular amyloidosis is believed to be a novel therapeutic approach not only for CAA but also for dementia.

### 2.2. Strategies to Tackle Cerebral Amyloid Angiopathy

Overproduction of Aβ or failure to eliminate it results in its accumulation; several investigations regarding sporadic AD and CAA have shown that the latter is critical [14,15], suggesting that promoting Aβ clearance would be a therapeutic approach for CAA [16]. The necessity of promoting Aβ clearance has been demonstrated in a clinical trial of Aβ immunization. In AN-1792-vaccinated AD patients, parenchymal Aβ plaques diminished, and cerebrovascular Aβ accumulation and CAA deteriorated [17,18]; this may be explained by the excessive antibody-solubilized senile plaque Aβ that is redeposited in the cerebral vasculature [19,20]. Aβ distribution in CAA as well as vaccinated AD cases closely corresponds to the intramural periarterial drainage (IPAD) route, which is one of the major systems related to Aβ elimination [21]. The IPAD route is also termed perivascular lymphatic drainage [22]. Interstitial fluid (ISF) and solutes including Aβ are believed to be cleared from the gray matter through the IPAD route, which is a space between two basement membranes in the walls of the cerebral capillaries and arteries [23,24]. IPAD has been shown to be impaired in the aging mouse brain and in the presence of CAA [25]. Furthermore, we have previously demonstrated that cilostazol, a selective inhibitor of type-3 phosphodiesterase, promotes IPAD, resulting in the maintenance of vascular integrity, amelioration of Aβ deposits, and prevention of cognitive decline [26]. Promotion of IPAD is now considered as a promising strategy to treat CAA.

Inhibiting Aβ assembly would be another potential approach to treat CAA as well as AD. The Aβ protein is secreted from an Aβ precursor protein (APP) through sequential proteolytic processing by β- and γ-secretases [27]. There are two major forms of Aβ: Aβ_40_ and Aβ_42_; Aβ_42_ is more neurotoxic because of its higher hydrophobicity, which subsequently results in faster aggregation. Aβ_42_ accumulation is marked in parenchymal senile plaques in AD brains, whereas Aβ_40_ accumulation is abundant in CAA and is closely related to cerebrovascular dysfunction [28,29,30]. Both Aβ molecules are generated in neurons as a monomer [31] and then aggregate to form oligomers, protofibrils, and fibrils through intermolecular β-sheet formation, thereby exhibiting various degrees of toxicity [32]. Aβ fibril formation is mediated by a nuclear-dependent polymerization process, which comprises nuclear and elongation phases [33,34,35].

Importantly, not only insoluble Aβ fibrils but also soluble Aβ including oligomers and protofibrils can induce neuronal and cerebrovascular injuries. Cerebrovascular dysfunction has been observed even before the appearance of insoluble Aβ accumulation around vessels in rodent models [36,37], which suggests that soluble Aβ is likely to impair cerebrovascular integrity and cognitive function in early stages of AD [37,38,39]. Therefore, the prevention of Aβ aggregation, especially in the early phase of CAA, is needed.

## 3. Therapeutic Potentials of Taxifolin for Cerebral Amyloid Angiopathy and Alzheimer’s Disease

### 3.1. Therapeutic Effects of Taxifolin on Cerebral Amyloid Angiopathy

Despite studies demonstrating pathological roles of Aβ in CAA, no effective treatments for CAA have been established. However, accumulating evidence has suggested the emerging effectiveness of taxifolin as a potential agent in the prevention and treatment of Aβ-associated cognitive dysfunction. Historically, oxidative stress has been reportedly closely implicated in the pathogenesis of age-related cognitive dysfunction, because the rate of oxidative metabolism is higher in the central nervous system than that in other tissues, and oxidative damage in the brain progresses with aging [40]. Furthermore, Aβ is also involved in the production of reactive oxygen species and causes neuronal dysfunction [33]. Therefore, dietary intervention with antioxidants has been expected to alleviate oxidative damage in the brain [40], thereby leading to reduced risk of cognitive dysfunction. Based on this possibility, extensive studies extracted a diverse array of compounds from various plants, characterized them, and addressed the potential neuroprotective effects of various antioxidants. During the course of these researches, taxifolin, a bioactive constituent of various plants, including onions, milk thistle, French maritime bark, and Douglas fir bark [41,42], was found and has become a topic of a great interest as a potential novel therapeutic target. Its biochemical and safety profiles have already been established [43,44]. Taxifolin is known to possess multiple pharmacological actions, such as anti-oxidation, advanced glycation end products (AGE) formation suppression, and mitochondrial protection, and has received increasing attention because of its potential efficacy in the treatment of various diseases including malignancies, cardiovascular diseases, chronic hepatitis, hyperlipidemia, and neurocognitive disorders [45]. 

We have recently addressed the potential therapeutic effects of taxifolin using in vitro and in vivo approaches and provided the first evidence delineating the novel beneficial effects of taxifolin on CAA [45]. The thioflavin T fluorescence assay and transmission electron microscopy imaging performed by us revealed that the addition of taxifolin to an Aβ_40_ solution significantly inhibited the aggregation of Aβ_40_ in vitro, indicating a novel suppressive effect of taxifolin on Aβ_40_ fibril formation [45]. Furthermore, we investigated the inhibitory effects of taxifolin on Aβ_40_ fibril formation in vivo using a mouse model of CAA, which expresses the human *APP* gene with Swedish/Dutch/Iowa triple mutations in neurons and also exhibits vasculotropic dominant accumulations of Aβ_40_ with respect to Aβ_42_ [45,46]. Quantitative analyses using filter trap assay and enzyme-linked immunosorbent assay showed that the cerebral levels of Aβ oligomers were decreased in the taxifolin group mice, which were fed taxifolin-containing chow, compared with the control group mice, which were fed standard chow. Therefore, these findings indicate that orally administered taxifolin has a novel preventive effect on Aβ_40_ fibril formation in the brains of CAA model mice [45].

We further addressed the effects of taxifolin on the pathogenesis of CAA using these mice [45]. The elimination half-life of taxifolin was found to be less than 1 h; thus, only a relatively small amount of taxifolin could pass the blood–brain barrier. Experiments to assess spatial learning and reference memory revealed that taxifolin significantly suppressed cognitive impairment in these mice compared with controls. As expected from taxifolin’s inhibitory effects on Aβ_40_ fibril formation, immunohistochemical analysis showed that it reduced the cerebrovascular accumulation of Aβ_40_ in CAA model mice compared with controls. Furthermore, laser speckle flowmetry indicated that taxifolin significantly restored the reduced cerebral blood flow in CAA model mice. Notably, in conjunction with the reduced cerebral Aβ oligomer levels and improved cerebral blood flow, blood Aβ_40_ levels were elevated in the taxifolin group mice compared with controls, suggesting that taxifolin facilitated the clearance of Aβ_40_ from the brain into systemic circulation; this would lead to a neuroprotective effect, contributing to cognitive impairment prevention [45].

### 3.2. Inhibitory Effects of Taxifolin on Amyloid-β_42_ Fibril Formation

Regarding Aβ_42_ fibril formation, which is closely implicated in AD pathogenesis, a previous meticulous study analyzed the effects of taxifolin on Aβ_42_ aggregation and β-sheet formation using wild-type Aβ_42_ or mutant Aβ_42_ carrying substituted amino acids [34]. The results demonstrated a novel mechanism of action of taxifolin in the inhibition of Aβ_42_ aggregation. The mechanism is related to the chemical structure of taxifolin: a catechol-type flavonoid, which possesses 3′,4′-dihydroxyl groups on the B-ring [34]. The catechol structure of taxifolin first autoxidizes and then forms *o*-quinone on the B-ring. This oxidized form, in turn, reacts with Aβ_42_ by targeting Lys16 and/or Lys28 of Aβ_42_, resulting in the production of Aβ_42_–taxifolin adducts. Importantly, Lys16 and Lys28 are located in the intermolecular β-sheet region of Aβ_42_. Therefore, the Aβ_42_–taxifolin adduct formation contributes to the inhibition as well as to the destabilization of Aβ_42_ aggregation, suppressing the elongation phase rather than the nucleation phase in the process of Aβ_42_ fibril formation [34].

### 3.3. Suppressive Effects of Taxifolin on Neuronal Amyloid-β Production

In addition to the biochemical properties of taxifolin that play a role in suppressing Aβ_42_ fibril formation, it is of importance to discuss the physiological significance of taxifolin in the prevention and/or treatment of cognitive impairment. Sequential cleavage of APP by secretases generates Aβ; the rate-limiting step in this process is the cleavage by β-site secretase enzyme (BACE1) [47,48,49]. As the expression levels and activity of BACE1 are elevated in the brains of AD patients [50,51], there is a possibility that Aβ production and fibril formation are enhanced in these patients. Several studies have addressed the underlying mechanisms regulating BACE1 gene expression [48,52,53]. Aβ_42_ induces the activation of JAK2 signaling pathway, which then mediates the activation of STAT3 signaling pathway [48]. The elevated STAT3 signaling, in turn, activates NF-κB signaling, which enhances the promoter activity of BACE1, thereby upregulating BACE1 transcription [48,52,53]. These signaling cascades promote amyloidogenesis, leading to neuronal injury and cognitive dysfunction.

To address the physiological significance of taxifolin in the suppression of cognitive decline, a study examined whether taxifolin is involved in attenuating signaling pathways for BACE1 expression using a mouse neuroblastoma N2a Swe cell line [48]. This cell line carries a human *APP* Swedish mutation and, when activated, the cells overexpress the gene, producing Aβ. Biochemical and immunocytochemical analyses revealed that the addition of taxifolin to the in vitro culture of these cells upregulated both the expression and the activity levels of SIRT1 [48], a deacetylase involved in the growth, differentiation, and survival of neurons [54]. Furthermore, the taxifolin-stimulated SIRT1 pathway reduced the activation of STAT3 signaling pathway, thereby downregulating BACE1 expression [48]. Together, these studies suggest novel functions of taxifolin besides the prevention of Aβ_42_ aggregation: taxifolin exhibits suppressive effects on neuronal Aβ production and subsequent Aβ fibril formation through reduction of BACE1 levels by stimulating SIRT1-mediated inhibition of STAT3 signaling pathway. Notably, the authors further demonstrated that cilostazol also exhibits beneficial effects on N2a cells, as observed with taxifolin treatment, by activating the SIRT1 pathway, alleviating the STAT3 pathway, downregulating BACE1 expression, and reducing Aβ production [48]. In particular, their finding that concurrent treatment with taxifolin and cilostazol results in synergistic suppressive effects on Aβ production and on neuronal cell death suggests novel potential therapeutic strategies for CAA as well as AD. In addition, the SIRT1 pathway stimulated by taxifolin and cilostazol might contribute to neurogenesis and cognitive function by potentially upregulating neuroprotective factors such as brain-derived neurotrophic factor [54].

### 3.4. Potential Therapeutic Effects of Taxifolin on Alzheimer’s Disease

Inflammation in the brain has been highly implicated in the pathogenesis of AD through the acceleration of amyloidosis [55] and neuronal cell death [48,56]. Studies have reported that in neurons, the activation of a proinflammatory mediator cytosolic phospholipase A_2_ (cPLA_2_) contributes to age-associated cognitive impairment [57] as well as AD pathogenesis [58,59]. Aβ_42_ can activate cPLA_2_ [60,61], which is responsible for the main enzymatic process of metabolizing arachidonic acid; this ultimately results in the production of prostaglandin E_2_ (PGE_2_), which is a neuroinflammatory molecule [42,61,62]. Both cPLA_2_ and PGE_2_ have been reported to cause synapse damage [63].

A recent study has investigated the effects of taxifolin on cPLA_2_-related inflammatory pathway and Aβ-induced neurotoxicity using the human neuroblastoma SH-SY5Y cell line and mouse primary hippocampal neurons [42]. Biochemical analysis revealed that the in vitro treatment of neurons with Aβ_42_ resulted in elevated levels of both cPLA_2_ and PGE_2_. Furthermore, live cell imaging showed that incubation with Aβ_42_ inhibited the formation of neuronal dendritic filopodia and dendritic spines. In contrast, the addition of taxifolin to these cultures seemed to combat Aβ_42_-induced neurotoxicity; taxifolin significantly prevented the increase in cPLA_2_ and PGE_2_ levels as well as the inhibition of dendritic filopodia and dendritic spines formation in neurons incubated with Aβ_42_. These data suggest that taxifolin exhibits neuroprotective effects besides its suppressive effects on Aβ production through the downregulation of BACE1 expression [48].

Using a mouse model of AD based on the hippocampal injection of Aβ_42_, the authors further examined the effects of intraperitoneal administration of taxifolin on the levels of cPLA2 and of the synaptic marker post-synaptic density protein-95 (PSD-95) and on cognitive function [42]. In line with the findings of the in vitro experiments, taxifolin suppressed the increase in cPLA2 and PGE2 levels in the hippocampus in Aβ_42_-injected mice. Moreover, Aβ_42_ injection reduced PSD-95 levels in the hippocampus, but taxifolin treatment significantly suppressed these reductions. Furthermore, animal experiments designed to test recognition and spatial memories reported that Aβ_42_-injected mice exhibited deficits in cognitive function, whereas taxifolin treatment improved this cognitive impairment. These results suggest that taxifolin exhibits suppressive effects on cognitive impairment in the preclinical settings of AD, potentially through pleiotropic functions including inhibition of Aβ_42_ fibril formation [34], suppression of Aβ_42_ production [48], and/or alleviation of Aβ_42_-induced neurotoxicity [42].

## 4. Therapeutic Potentials of Taxifolin for Metabolic Diseases with A High Risk for Neurodegenerative Diseases

### 4.1. Effects of Taxifolin on Diabetes

Epidemiological studies have reported diabetes to be a high-risk factor for dementia, including AD and vascular dementia [64,65,66]. Potential mechanisms underlying diabetes-related dementia include multifactorial pathways such as Aβ accumulation, neuroinflammation, small vessel infarcts, and neurodegeneration in the brain [64,65,66,67,68]. Accordingly, prevention and treatment of diabetes is critical to reduce the risk of development and progression of dementia.

Detailed findings regarding the effects of taxifolin on diabetes are limited, but a recent study has demonstrated the anti-diabetic effects of taxifolin and its mechanisms of action through in vivo and in silico approaches [69]. The authors used a rat model of diabetes in which pancreatic β-cells were depleted by intraperitoneal injection of alloxan. They found that taxifolin administration via an intragastric route significantly reduced blood glucose levels in the diabetic rats compared with controls (without taxifolin). To elucidate the underlying mechanisms of the hypoglycemic effects of taxifolin, the authors next examined the effects of taxifolin on α-amylase, a carbohydrate-metabolizing enzyme that elevates blood glucose levels; inhibition of α-amylase is effective in the treatment of diabetes [69,70]. Taxifolin treatment significantly reduced serum amylase activity in diabetic rats compared with controls, consistent with its glucose-lowering effects. These studies suggest that taxifolin exhibits hypoglycemic effects through the reduction of α-amylase activity in diabetic rats [69].

The authors further addressed the potential direct action of taxifolin on α-amylase with computational and docking studies, comprising ligand–receptor docking studies, free-energy calculations, and molecular dynamics simulations [69]. In the flexible docking simulations, the authors selected the bioinformatically determined best-docked poses of the taxifolin–α-amylase complex and analyzed the binding modes of taxifolin with α-amylase. The analysis revealed that taxifolin interacts with the residues Trp59, Tyr62, Glu233, and Asp300 present at the active site of α-amylase through a π–π interaction with the benzene rings of Trp59 and Tyr62 and an H-bond interaction with Glu233 and Asp300. In addition, using a molecular mechanics-based scoring method for binding free-energy calculation, the authors showed that van der Waals and nonpolar solvation-free energies also contribute to the binding affinity of taxifolin for α-amylase. Furthermore, the authors examined the dynamic behavior of the taxifolin–α-amylase complex through molecular dynamics simulations, considering the potential effects of solvent, temperature, and pressure on the complex formation, and confirmed the stable conformation of taxifolin at the active site of α-amylase. Accordingly, these in vivo and in silico findings indicate that taxifolin binds to the active site of α-amylase and inhibits its activity, thus leading to improvement of hyperglycemia [69].

### 4.2. Effects of Taxifolin on Diabetic Nephropathy

Diabetic nephropathy is a serious diabetic complication [71], and chronic kidney diseases (CKDs) are epidemiologically a high-risk factor for dementia [72,73,74]. Reportedly, in a mouse model of CKD, chronic renal dysfunction resulted in elevated oxidative stress levels in the brain, leading to cognitive impairment [75]. Thus, the prevention and improvement of CKDs would contribute to reduce the dementia risk.

Recent studies have reported the novel renal protective effects of taxifolin using a rat model of diabetes, which was developed through pancreatic β-cell depletion with an intraperitoneal streptozotocin injection [71,76]. Taxifolin treatment significantly improved the renal function profiles in diabetic rats compared with controls, in parallel with improved glucose metabolism [71,76]. Consistent with these results, further histological analyses revealed that taxifolin suppressed necrotic cell death in the renal tissue [76] and alleviated renal fibrosis by inhibiting extracellular matrix accumulation and mesangial matrix expansion [71]. Furthermore, biochemical analyses showed that taxifolin reduced the activation of high-glucose-stimulated proinflammatory pathways in rat and human kidney cell lines [71] as well as in renal tissue from diabetic rats [76]. Taxifolin also reduced the levels of reactive oxygen species produced by these kidney cell lines, which were stimulated with high glucose [71]. These findings indicate the potential renal protective effects of taxifolin in diabetic conditions, further supporting its potential beneficial effects on dementia.

### 4.3. Effects of Taxifolin on Obesity

Obesity has been implicated in the development of dementia in later life [77]; however, a recent study has reported an inverse association between body mass index and dementia incidence [78]. Thus, the potential effect of obesity on dementia incidence remains controversial. Obesity is a high-risk factor for diabetes, cardiovascular diseases, and CKDs [79], which, in turn, are risk factors for dementia [64,65,66,72,73,74,80]. Therefore, improvement in obesity would be beneficial for reducing dementia risk.

Recent reports have demonstrated novel roles of taxifolin in improving obesity [81,82]. The authors analyzed the effects of orally administered taxifolin on a rat model of diet-induced obesity (high-fat diet). The taxifolin group showed significant reductions in body weight and serum cholesterol and triglycerides levels compared with the controls (without taxifolin treatment) [81]. Taxifolin also improved hyperglycemia and insulin resistance as well as oxidative stress levels [82]. Furthermore, it elevated gene expression levels of mitochondrial uncoupling protein-1 and carnitine palmitoyltransferase I, markers for fat oxidation and energy expenditure of the energy-consuming brown adipose tissue [82]. Together, these findings suggest a novel anti-obesity effect of taxifolin, potentially mediated through an improvement of glucose and lipid metabolism as well as of energy homeostasis [81,82], although the mechanistic details underlying these effects remain to be elucidated.

## 5. Future Perspectives

**Figure 1:**
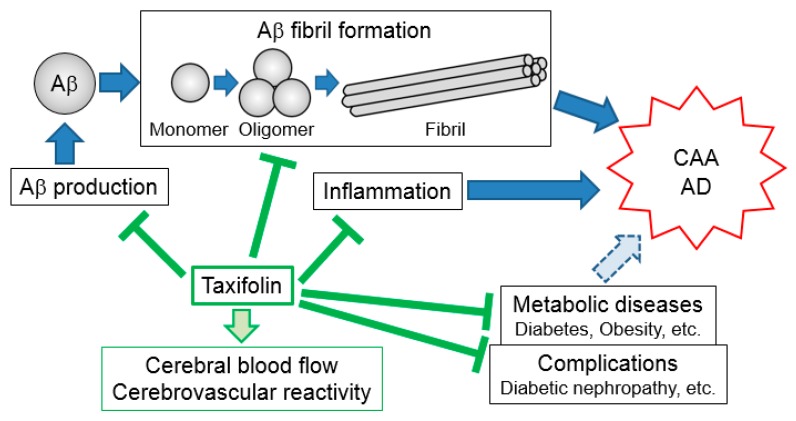
Pleiotropic beneficial effects of taxifolin. Taxifolin suppresses Aβ production, fibril formation, and neuroinflammation in the brain. It also ameliorates cerebrovascular dysfunction. Furthermore, taxifolin beneficially affects certain metabolic diseases with a high risk for neurodegenerative diseases and their complications. These direct and indirect effects of taxifolin would contribute to preventing and/or treating Aβ-associated cognitive dysfunction including CAA and AD. Aβ: amyloid-β; CAA: cerebral amyloid angiopathy; AD: Alzheimer’s disease.

Aβ fibril formation plays pathological roles in neuronal injury. In particular, Aβ_40_ and Aβ_42_ aggregates are mainly implicated in the pathogenesis of CAA and AD, respectively. Recent advances in taxifolin research have provided novel insights into its pleiotropic beneficial actions, which include inhibition of Aβ fibril formation [34,45], suppression of Aβ production [48], and facilitation of Aβ clearance [45], contributing to the suppression of the development and progression of Aβ-associated cognitive dysfunction. Furthermore, taxifolin has been shown to improve metabolic diseases with a high risk for neurodegenerative diseases and their complications [69,71,76,81,82]. These findings suggest that taxifolin is a key molecule in the prevention and treatment of cognitive dysfunction as well as metabolic diseases with an increased risk for neurodegenerative diseases (Figure 1, Table 1).

Emerging research has indicated physiological roles of taxifolin, which has raised new issues for further investigation. One such issue is to identify the molecular targets and/or receptors of taxifolin. In addition to its binding to Aβ and α-amylase [34,45,69], taxifolin modulates the phenotypes of cells and animals [42,45,48,69,71,76,81,82], suggesting that taxifolin also potentially binds to receptors/transcription factors which would subsequently affect intracellular signal transduction and gene expression profiles. Determining the molecular mechanisms of taxifolin-stimulated signaling pathways would provide significant clues for the identification of novel molecular targets to prevent and treat cognitive dysfunction and metabolic diseases.

Another important issue is the modification and/or synthesis of novel bioactive molecules based on taxifolin. In a study, 191 taxifolin metabolites were detected in various tissues of taxifolin-fed rats [41], and it is possible that each of these metabolites has a different functional significance. Accordingly, further bioinformatical and experimental approaches using these data will lead to the development of novel bioactive therapeutic molecules which exhibit improved taxifolin-related cell type- or tissue type-specific activities, depending on the disease type.

Finally, issues yet to be addressed include the clinical significance of taxifolin. Future interventional studies in humans should aim to determine the effects of taxifolin on prevention and treatment of cognitive impairment as well as metabolic diseases; these studies would contribute to developing novel strategies for reducing disease risk. In this respect, a double-blind, placebo-controlled, randomized early phase II study is in progress (“Cilostazol for prevention of conversion from mild cognitive impairment (MCI) to Dementia (COMCID) study), which is aimed at evaluating the efficacy and safety of cilostazol in patients with mild cognitive impairment [83]. The beneficial effects of cilostazol on neuronal cell lines have been found to be similar to those of taxifolin [48], even though they were based on in vitro results; thus, the findings of the COMCID study will allow us to gain insights into the clinical potential of taxifolin. Furthermore, investigating the efficacy and safety of the co-treatment with taxifolin and cilostazol would help establish novel therapeutic strategies for CAA and AD.

In conclusion, recent studies on taxifolin have provided a better understanding of its mechanisms of action and have highlighted its physiological significance and therapeutic potential for Aβ-related cognitive impairment as well as for metabolic diseases with an increased risk for neurodegenerative diseases. It should be noted that there has been no new drug approved for the treatment of AD over the past 15 years, despite extensive research and clinical trials with candidate drugs targeting Aβ accumulation [84]. This may increase the possibility that Aβ accumulation is a by-product of the AD process rather than a cause, thereby suggesting the importance to address alternative hypotheses to the amyloid cascade hypothesis [84]. However, Aβ has been recently associated with cytotoxicity; therefore, Aβ should be targeted for the treatment of AD and CAA. In this respect, findings from Aβ immunization studies in which antibody-solubilized Aβ from the senile plaque redeposited in the cerebral vasculature and exacerbated CAA [17,18,19,20], as described earlier, suggest that not only prevention of Aβ aggregation but also efficient clearance of Aβ should be a key strategy for the treatment of AD and CAA [16]. Furthermore, other cytotoxic mediators in the brain include proinflammatory cytokines and reactive oxygen species, and identifying these factors would also be useful for the development of treatments for AD and CAA. In this context, this present study provides the first evidence that orally administered taxifolin suppressed Aβ expression, reduced proinflammatory cytokine levels, alleviated oxidative tissue damage, and reduced the markers of apoptotic cell death in the brain of CAA model mice [85]. Together with results demonstrating the effective clearance of Aβ [44], these findings highlight the use of taxifolin as a potential novel therapeutic target for AD and CAA. Future basic and clinical studies aimed at clarifying the molecular mechanisms underlying taxifolin’s pleiotropic beneficial effects would open new avenues for preemptive medicine for dementia and address its causative metabolic dysfunctions.

## Figures and Tables

**Table 1:** Favorable effects of taxifolin on AD and CAA.

Pharmacological Effects	Targets	Mechanisms
Suppressing Aβ production	Neuron	Reduction of BACE1 levels
Inhibiting Aβ aggregation	Lys residues of Aβ	Aβ–taxifolin adduct formation
Anti-inflammation	Neuron	Reduction of cPLA_2_ and PGE_2_ levels
Increasing CBF and CVR	Vascular endothelial and/or mural cells	Amelioration of Aβ toxicity Anti-oxidation Anti-glycation
Reducing hyperglycemia	α-amylase	Taxifolin–α-amylase complex
Reducing body weight	Brown adipose tissue	Increased energy expenditure
Renal protective effects in diabetic conditions	Renal tissue	Anti-fibrosis Anti-oxidation

BACE1: β-site secretase enzyme; CBF: cerebral blood flow; CVR: cerebrovascular reactivity; cPLA_2_: cytosolic phospholipase A2; PGE_2_; prostaglandin E_2_.

## Abbreviations

AD	Alzheimer’s disease
Aβ	Amyloid-β
CAA	Cerebral amyloid angiopathy
IPAD	Intramural periarterial drainage
